# Need for Nurse Practitioner Fellowships in Ophthalmology in the USA

**DOI:** 10.18502/jovr.v16i1.8257

**Published:** 2021-01-20

**Authors:** Vishwani Persaud-Sharma, Mary A. Hooshmand

**Affiliations:** ^1^School of Nursing and Health Studies, University of Miami, Miami, FL, USA

**Keywords:** Fellowships, Nurse Practitioner, Ophthalmology, Post-graduate Training, Residency, United States

## Abstract

Medical attention to vision impairment and associated eye care complications are a vital component of daily living and overall well-being. In the United States today, the physician to patient deficit places great strain on the availability of medical attention tenable to patients nationwide; in terms of specialty medicine, this deficit is even more widespread. The field of ophthalmology faced the same physician to patient deficit in 2020, a grim reality that has left many states void of ophthalmic care, rending millions of aging individuals without domestic eye care. The implementation of trained, ophthalmic nurse practitioners (NPs) can fill the needs of this deficit; however, efficient, accredited, and board-approved American ophthalmic fellowships and residencies that secure proper ophthalmic NP transitions from academia to clinical practice are non-existent. Though scant, evidence-based literature presents sound findings that support the efficacy and benefit for superior patient outcomes with care provided by ophthalmic-trained NPs, offering a viable, long-term solution to the need for ophthalmic medical providers across all states without mitigating patient care, emphasizing the great need for the implementation of ophthalmic NP residencies and fellowships to ensure the continuity of impeccable ophthalmic care for all populations.

##  INTRODUCTION

To date, there exists a severe shortage of eye care providers that perpetuates unnecessary vision impairment and blindness in developing and developed countries worldwide.^[1]^ In the United States (US) and according to the Association of American Medical Colleges (AAMC), America will observe a physician shortage of approximately 122,000 by the year 2032.^[2]^ The current physician shortage is pragmatic in primary care services, which is projected to rise due to the ever-growing population and increasing population age, estimated to account for 81% of the total population from 2010 to 2020.^[3]^ Specialty shortages also form a significant disparity in provider healthcare, where the projected medical specialist dearth rates are projected to fall between 1,900 and 12,100; the projected surgical specialist shortage is approximated to fall between 14,300 and 23,400, while other specialists like neurologist, pathologists, psychiatrists, and radiology specialists can anticipate a shortage of 20,600 to 39,100 by the fiscal year of 2032.^[2]^ Specifically, a total deficit of 45,400 primary care physicians and 46,100 medical specialists, a grand total of 91,500 medical doctors will be needed in the fiscal year of 2020 alone.^[4]^ Recent data acquired in 2020, post onset of the COVID-19 pandemic, projects the physician shortage to dramatically worsen by 2033; in (i) primary care, the physician shortage will range from 21,400 to 55,200 physicians, (ii) in non-primary care specialties, the shortage will fall between 33,700 and 86,700 physicians, (iii) in surgical specialties, the shortage will be between 17,100 and 28,700 physicians, (iv) in medical specialties, the shortage will be between 9,300 and 17,800 physicians, and finally (v) in other specialties such as radiology, pathology, and psychiatry, there will be a 17,100 to 41,900 physician shortage.^[5,43]^ The cumulative need for physicians in the US emphasizes the roles of primary care Nurse Practitioner (NP) and Physician Assistant (PA) workforces, which is anticipated to grow at a greater rate compared to physician supply; the supply of the primary care NPs is expected to see a 30% increase, where primary care PAs are expected to increase by 58% through 2020.^[3]^ More recently, the 2019 role of the NP has grown by over 270,000 in the US, as patients are now benefiting more than ever from comprehensive, high-quality, patient-centered healthcare services governed and provided exclusively by NPs.^[2]^ Additionally, an AAMC study analyzing the effective use of the NP and PA workforce to compensate for the growing healthcare provider paucity projected a potential physician shortage decrease of 42,600 to 121,300 by 2030.^[6]^

Through the effective integration of Advanced Practice Clinicians (APCs) in the medical field, the projected deficit of primary care physicians can decrease to 6,400.^[3]^ Studies conducted by Spetz *et al* ^[7]^ and Hoff *et al* ^[8]^ illustrate the positive patient perception and care provided by APCs in diverse patient populations including primary care and medical specialties. Comparative studies conducted by Jiao *et al* ^[9]^ detail the relative comparability of ambulatory prescribing among physicians and APCs alike. While Hooker *et al* ^[10]^ delineates the different characteristics among APCs, NPs have been specifically noted to fully utilize their APC skills, practice to the maximum capacity of their legal scope, are satisfied with their careers, and plan to stay in their jobs log-term, all while reporting greater practice autonomies.^[7]^ In specialty fields, trends assessed by Ray *et al* ^[11]^ acknowledge the lack of research addressing APC involvement in medical specialties. It was concluded that patient visits involving APCs in surgical and medical specialties increased from 3.3% between 2001 and 2003 to 6.9% between 2010 and 2013, lending credit to the effectiveness and increasing need of APC visits in specialty medicine.^[11,12]^ Effective use of APC practice in specialties are further bolstered and defined by the implementation of APC fellowships and residencies, facilitating adequate transition into specialized medical care. Additionally, education and training not only strengthen and develop the capabilities of global eye healthcare and the World Health Organization Development Goals in a sustainable way, but they also direct focus and bolster the skills and efficacy of ophthalmic providers in the US to ensure quality and precision care, while addressing the need for qualified and superiorly trained specialty eye care providers, a void that can be fulfilled by ophthalmic NPs.^[1]^ The purpose of this article is to draw attention to the need for Nurse Practitioner Fellowships in the US with specific attention to NP fellowships and residencies in specialty medicine like ophthalmology.

### The Importance of NP Fellowships in the US 

The terms fellowship and residency are used synonymously in APC literature.^[13]^ Generally, medical and pharmacy fellowship and residency programs serve to provide adequate transition of the new healthcare practitioner from academia to clinical practice; the APC transition is no different, especially in specialty practices.^[14]^ Both PA and NP accreditation bodies have established postgraduate training models governed by the (i) Accreditation Review Commission on Education for Physician Assistant (ARC-PA) and

(ii) the American Nurses Credentialing Center (ANCC) and National Nurse Practitioner Residency and Fellowship Training Consortium, respectively.^[15]^ As of 2007, 60 APC postgraduate training programs were functional in the US, with primary attention toward surgical specialties.^[15]^

As of 2019, there existed 145,585 certified NPs in the US; clinical areas of field certification include acute care, adult care, adult psychiatric-mental health, gerontology acute care, gerontology primary care, diabetes management, family medicine, pediatrics, psychiatric-mental health across lifespan, school NPs, and emergency medicine.^[16]^ While the NP scope of practice is discussed at length by Hudspeth and Klein,^[17]^ it is important to underscore the recent legislative changes that now enable more NPs to practice autonomously in the majority of the states in the US. According to Park *et al*,^[18]^ greater NP practice autonomy was attributed to full, independent prescriptive authority, whereas having independence governing medical diagnoses and treatment regimens only moderately affected prescriptive independence. Such results indicate that expanded state NP practice regulations correlated with an increase in NP supply and greater access to care among rural and underserved populations deprived of a decrease in care quality.^[19]^ Recent literature directly affirms and correlates NP autonomy and favorable relationships with leadership improves teamwork in the clinical provider workforce.^[42]^ Additionally, there is a clear correlation between interdisciplinary teams and better patient outcomes; interdisciplinary teams within clinical practice effectively facilitates teamwork, inter-collegiality, and superior clinical provider and leadership relationships, which yield better care outcomes.^[21,22,23]^ Finally, a study conducted by Poghosyan *et al* ^[20]^ also affirms and provides tangible evidence that NP–physician teamwork directly affected clinician job satisfaction, intent to leave, and perceived quality of care within a given medical practice.

Though there are many facilitators and barriers that both aid and negate effective and confident NP workforce transition, the implementation of NP fellowships can serve as a platform to sustain effective shifts from the academic to clinical platform; facilitators like the establishment of mentorship, social support, meaningful work, and work–life balance as well as barriers to NP workforce transition such as lack of support, role ambiguity, and workload exists have been founded to impede and bolster this process, a challenge that can be resolved by NP fellowship implementation.^[24]^ According to Bryant and Parker,^[25]^ participation in a nurse practitioner fellowship instills greater confidence, job satisfaction, and increased job retention through the transition from novice to expert clinician; as a result, continued provision of NP fellowships facilitate superior clinical practice leading to greater patient outcomes provided by NPs. While NPs are noted to deliver cost-effective, high-quality medical care that addresses the need for medical providers, graduate education often lacks specialized postgraduate fellowships, resulting in the acquisition of on-the-job training.^[26]^ With emerging research highlighting the need for NP fellowships across US specialty disciplines, Kesten and El-Banna^[27]^ found that over 90% of program directors state an increase in NP recruitment and retention following NP fellowship implementation. Additionally, the majority of decision-makers favor NP fellowship implementation with few to no barriers and 84% of physician and administrative support and favor fellowship/residency acquisition.^[27]^

### NP Fellowships and Residencies in Specialties – What is known?

As of 2016, more than 30 postgraduate fellowships are available for masters and doctorly prepared NPs to enhance their teaching, clinical outcomes, advocacy, and research abilities.^[13]^ A total of 68 active NP fellowships and residencies were identified by Martsolf *et al* ^[28]^ in the US, where 45.6% of programs were self-defined as residencies and 54.5% self-defined as fellowship programs. The average postgraduate NP fellowships varied from 12 to 24 months in duration and offered predominantly full-time status with competitive salaries and benefits.^[28]^ NP fellowship salaries averaged $60,000 USD, with the highest noted at over $100,000 USD; some programs reported a salary of <$50,000 USD, whereas other fell within the $50,000 to $60,000 USD range.^[28]^

In terms of admission requirements, 79.4% of the 68 NP postgraduate programs required a state NP license, 67.7% required a discipline-specific certificate, 51.5% targeted new graduates, 22.1% required additional certification specific to the program, 51.5% required an NP specific degree such as pediatric or family NP, and 17.7% required a Drug Enforcement Agency number (DEA).^[28]^ Performance and effect of increased ability, patient satisfaction, and quality of care are further evaluated in detail by Hoff *et al* ^[8]^, Kesten and El-Banna,^[27]^ Sciacca and Reville,^[13]^ and Spetz *et al*.^[7]^ Examples of recent specialty NP fellowships successfully implemented within the last five years are depicted in Table I, in the fields of oncology,^[26]^ palliative care,^[29]^ emergency medicine,^[30]^ and neurology.^[31]^

**Table 1:** Examples of US NP fellowships across medical specialties in the past 5 years


**Citation**	**Country**	**Program Type**	**Model**	**Aim**	**Outcome**
Alencar *et al*, 2018^[26]^	USA	ARNP Oncology Fellowship	ARNP Model	Define the need for ARNP Fellowship in Oncology	1. Structured ARNP fellowships in oncology facilitate training, mentorship, and retention 2. Implementing new NP oncology fellowship lead to increased patient care, job and staff satisfaction
Dahlin *et al*, 2019^[29]^	USA	Hospice & Palliative Care APRN Fellowship	HPNA APRN Fellowship Guidelines	Detail aspects of six Palliative APRN fellowships	1. APRN Fellowship improved patient outcomes
Hardeman & Hough, 2017^[31]^	USA	APRN and PA Fellowship in Neurology	ARNP and PA Model	Define need for advanced practice practitioner fellowship in Neurology	1. Need for APC in neurology backed by statistics that reflects high patient burden 2. APP Neuro fellowship will train, retain, and ease neuro clinician shortage 3. APP more cost-effective, better patient outcomes
Gaudio & Borensztein, 2018^[30]^	USA	ARNP Emergency Medicine Residency	ARNP Model	Define the need for ARNP Residency in Emergency Medicine	1. Increased ENP self and job satisfaction 2. Increased ENP competency 3. Stronger clinical foothold in EM

Predominant NP fellowship and residencies offered throughout the US to date are distributed disparately throughout each state, where some states do not offer any NP fellowship programs whatsoever. NP fellowships and residencies predominate in advanced practice, advanced practice nursing, acute adult care, cardiology, critical care, diabetes, dermatology, emergency medicine, family nurse practitioner, gastroenterology and hepatology, geriatric, neuroscience/neurology, oncology, orthopedic, palliative care, pediatrics, surgical, and wound reconstruction among other variations based on demographic and state need; however, there is no NP ophthalmology fellowship or residency available to date.^[32]^

### Defining the Need for NP Fellowships in Ophthalmology

As a clear delineation circumscribes the countless benefits provided by NP health services in the medical profession in terms of physician deficit burden, patient outcomes, and quality of care, clinical efficiency is bolstered through the implementation of NP fellowships, especially in specialty medicine.^[25,27]^ To date, there is minute to no literature that supports the need to establish an NP fellowship in the specialty field of ophthalmology.

### The Value of Advanced Practice Ophthalmology Nursing

While the physician to patient burden is prevalent in all medical disciplines, there is paralleled heightened urgency in the field of ophthalmology; by the year 2020 compared to 2000, the total population to ophthalmologist ratio has increased by 15% with a projected increase over time.^[33]^ Such a shift in demand can be largely attributed to the increase in the elderly population, who heavily rely on ophthalmic services, drawing attention to the need for additional ophthalmology health providers.^[33]^ As illustrated by Browning,^[33]^ there are three predominant methods to address the need gap in ophthalmology care, namely (i) increase the number of ophthalmology providers, (ii) enable current and future ophthalmologists to work more hours, or (iii) institute and effectively utilize APCs in the field of ophthalmology. Historically, an average of 52 PAs were employed by ophthalmologists by 1990; that number has since increased to 70 as of the fiscal year 2015.^[33]^ Established duties known to ophthalmology PAs include preoperative histories and physical exams for large cataract and refractive surgery; however, Browning^[33]^ states that PAs can do more such as take call, conduct clinical work-in visits, perform intravitreal injections (IVTs) for retinal specialties, and operate dry eye clinics. As effective as PA duties are in ophthalmology, the role of the NP is even more so, making NPs an invaluable addition to the field of ophthalmology.

From a financial perspective, Moore and Barr^[34]^ further define the potential resolution of bridging the ophthalmology physician deficit burden with the use of APCs, optometrists, faculty ophthalmologists, and resident ophthalmologists. Though a detailed overview approximated the average salary and benefit wages to be $126,797, $117,021, $338,233, and $71,210, respectively, the study concludes that while the use of ophthalmology residents to address the ophthalmologist shortage is more cost-effective, they do not directly produce work relative value; therefore, long-term implementation of resident ophthalmologists to address the need is not a viable long-term solution.^[34]^

Advanced practice NPs are educated to provide competent, independent, autonomous patient care; they have the ability to manage their own health clinics and provide adequate and efficient healthcare for their own governing patient populations.^[35]^ Advanced practice NPs have the ability to adjust, expand, and integrate practical skills, and evidence-based research into patient care regimes to meet the demands and expectations of patients, governing bodies, and stakeholders.^[35]^ In terms of ophthalmic medicine, the benefits of ophthalmology NP implementation is no different.

#### Ophthalmic NP Duties

Ophthalmology NPs have the ability to evaluate, diagnose, treat, and discharge patients with ocular disorders.^[35]^ They have the ability to manage care for referred patients from general and primary care providers, conduct baseline screenings, monitor disease development and outcomes, and treat chronic ocular conditions such as diabetic retinopathy, dry eyes, and glaucoma among other ocular disorders.^[35]^ In terms of surgical care, ophthalmic NPs can conduct initial, follow-up, and discharge assessments and education for ophthalmic surgery patients diagnosed with cataract among other ocular disorders; they can also manage care on a broad spectrum, from children to adults to the older adults.^[35]^ Additionally, ophthalmic NPs can perform minor ophthalmic procedures autonomously without physician supervision, such as adnexal surgery and assisting in ophthalmic surgeries like YAG laser capsulotomies.^[35]^

#### Tangible Evidence of Successful Ophthalmology NP Implementation

To date, there is currently one study that documents the successful implementation of a single PA into an ophthalmology consulting service in an academic setting; the purpose was to improve resident education with an outcome of improved ophthalmic resident education facilitated by a PA overall.^[36]^ The implementation of advanced practice NPs into an ophthalmology clinic dates back to 2007, a case study that documents an ophthalmology NP effectively providing NP-led consultation services to a diabetic retinopathy patient in Wales.^[37]^ Harty^[37]^ clearly delineates the value of the ophthalmic NPs in a patient's most vulnerable state and reiterates the fact that if no ophthalmic NP serves were provided, the patient would have suffered additional, unnecessary trauma and anguish potentially leading to blindness. A literature review complied by Drury *et al* ^[38]^ of Australia documents the effectiveness of advanced practice ophthalmology NPs, indicating that while the majority of nurse-led ophthalmology clinics are supervised by ophthalmologists, there are many autonomous clinical skills performed by the ophthalmic NP such as slit lamp examinations, fundus examinations via direct ophthalmoscope use, optic disc assessment, and anterior segment assessments.^[38]^ Additionally, Drury *et al* ^[38]^ highlighted the variability in ophthalmic NP training, stating that two documented studies delineated the training of ophthalmic NP-led clinics who held a Master's degree with postgraduate training in pharmacology and extensive anterior segment training. Such services are meant not to facilitate replacement of the ophthalmologist yet render adjunct ophthalmic services to shorten waiting lists and allow providers to spend more time caring for complex patient needs.^[38]^ Finally, in a Scottish study by Gallagher *et al*,^[39]^ an advanced ophthalmic NP delineated the effective and suitable implementation of ophthalmic NPs in IVT clinics given their training and experience; demonstrating NP expansion in the ophthalmic discipline in terms of IVT, macular assessment and follow-up, and effective patient care and outcomes for those diagnosed with age-related macular degeneration, macular edema-associated diabetic retinopathy, and retinal vein occlusion. Findings of the study indicate that most of the polled ophthalmic population found the delivery of IVT provided by an ophthalmic NP to be more educating, receptive to questions, and patient centered.^[39]^ Additionally, patients did not mind IVT delivery performance via a trained, ophthalmic NP versus a physician, and of those who objected to IVT via an NP over a physician cited concern for decreased training and experience to deal with consequential problems as the primary mode of concern.^[39]^

### SUMMARY: US Ophthalmology NP Fellowships, It Is Needed. What Now?

To date, there are no established ophthalmology NP fellowships recorded within the past 10 years in the US. Given the increasing physician deficit to increased population burden that is echoed in the discipline of ophthalmology, the time for APC implementation in ophthalmology has arrived.^[33]^ The importance of APC provider healthcare is boundless; with increased autonomy in the US for NPs across various states; NPs offer a cost-effective, efficient, and patient-centered option to providing medical care across demographics and socioeconomically challenged populations. In an effort to standardize and direct the role of the NP, the APRN Consensus Work Group and National State Boards of Nursing formed the 2008 Consensus Model, mandating NPs to obtain a proper education with a graduate degree or postgraduate certification from an accredited university among other requirements.^[12]^ Over time, NP schooling requirements, clinical knowledge, and patient practicums have developed more rigorously to ensure efficacy of care provided.

While the acquisition of postgraduate APC fellowships or residences are sparse, participation in accredited programs bolster the skillset, mental acuity, and evidence-based care provided to the given, served population. They function to bridge the gap in clinical practice among APCs.^[15]^ It is here that the APC learns to transition their academic knowledge to the clinical setting in a safe, supervised, and directed platform. Favorable outcomes of such programs have been noted to augment care, where patients feel reassurance in knowing that the APC underwent rigorous and accredited educational standards to ensure their privilege at the bedside as a medical care provider. As stated by Cosme,^[14]^ continued growth of residency and fellowship programs for APCs are needed in order to meet the growing demand of healthcare needs in terms of patient safety and decreased reimbursement; continued growth will safeguard increased self-reflection and drive research that will better both medicine and healthcare consumers as a whole. Additionally, participating in a postgraduate NP training program, residency, or fellowship aids in the creation of valuable members of the healthcare team that can function during rapid changes in the American healthcare system.^[13]^ Moreover, participating in APC postgraduate residencies or fellowships aids to calm the anxiety associated with the transition from academia to clinical practice, all while obtaining supervised training and expert mentorship.^[13]^

Current ophthalmology statistics underscore the need and shortage of ophthalmologists, where 61% of Americans had no ophthalmologist in 2011; a shift in population distribution toward an aging population surmises the need for ophthalmic services across the country.^[33]^ While there are many solutions to bridging the need for ophthalmic physicians such as working longer hours and expanding Medicare, the use of APCs can bridge the deficit.^[33]^ Although initial studies emphasized the efficacy of trained PAs in ophthalmology, NPs are equally if not more viable in terms of trainability, clinical experience, cost, and clinical background, making NPs highly suitable for ophthalmic care following the successful completion of an accredited ophthalmology residency or fellowship.

In terms of ophthalmology APCs, studies prove that successful postgraduate training for advanced practice NPs in the field of ophthalmology enable efficient patient care in the various aspects of ophthalmic care.^[37,38,39]^ As described by Drury *et al*,^[38]^ following NP ophthalmic-specific training, nurse-led ophthalmic clinics successfully functioned to enable NPs to complete common ophthalmic practice such as slit lamp exams, direct ophthalmoscope fundus examinations, optic disc assessments, and anterior segment assessments among other critical techniques and practices needed for independent ophthalmic assessment, care, and treatment. Additionally, ophthalmology simulations offer cost-effective, heighted accessibility, objective ophthalmology training outcomes, and improved patient safety initiates to effectively train APCs with specific attention to NPs in the specialty field of ophthalmology.^[1]^ As current practices in the US do not facilitate ophthalmic fellowships or residencies, the purpose of this article was to delineate the need and benefit for immediate implementation.

Although the NP workforce transition can be rigorous at times, there are many strategies that can facilitate the effective transition of the NP into a proper clinician and leadership role; self-initiative, mentorship, experiential learning, professional socialization, and interprofessional training are effective and proven methods that facilitate operative, sustainable, and substantial clinician–patient relationships in an effort to provide superior patient care, methods that are absolutely critical and effective in molding impeccable ophthalmic NPs.^[40,41]^ The primary objective of specialized postgraduate ophthalmic NP fellowships would be to educate and train NPs to be fast, logical thinkers under pressure and during emergent situations; decisions should compile assessment and utilization of prior studied information for accurate situation evaluation, all while rationalizing best patient outcomes, just as US physicians undergo in post-medical school residencies.^[35]^ As Martsolf *et al* ^[28]^ describes, the need to establish NP ophthalmology fellowships coincides with the Institute of Medicine's seminal report that urges the state boards of nursing, accrediting bodies, the federal government, and healthcare organizations to enact methods that support nurses' completion of a transition-to-practice program, such as a residency or fellowship, after completing prelicensure, advanced practice degrees, or when transitioning into new clinical practice areas. The need for ophthalmology NP fellowships in the US is clear; the time for establishment is now.

##  Financial Support and Sponsorship 

Nil.

##  Conflicts of Interest 

There are no conflicts of interest.
